# The brain–heart-immune axis: a vago-centric framework for predicting and enhancing resilient recovery in older surgery patients

**DOI:** 10.1186/s42234-024-00155-4

**Published:** 2024-09-02

**Authors:** Leah Acker, Kevin Xu, J. P. Ginsberg

**Affiliations:** 1 Department of Anesthesiology, Duke University School of Medicine, 136 Sands Building, 303 Research Drive, Durham, NC 27710 USA; 2 Department of Neurobiology, Duke University School of Medicine, Durham, NC USA; 3 Pratt School of Engineering, Duke University, Durham, NC USA; 4 Duke Center for the Study of Aging and Human Development, Durham, NC USA; 5 Claude D Pepper Older Americans Independence Center at Duke, Durham, NC USA; 6 Duke Center for Cognitive Neuroscience, Durham, NC USA; 7 William Jennings Bryan Dorn VA Healthcare System, Columbia, SC USA

**Keywords:** Brain–heart-immune axis (BHI-a), Neuro-immune modulation, Vagus nerve stimulation (VNS), Autonomic nervous system (ANS), Resilience, Aging, Delirium, Postoperative delirium (POD), Perioperative medicine, Heart-rate variability (HRV), Wearable devices

## Abstract

Nearly all geriatric surgical complications are studied in the context of a single organ system, e.g., cardiac complications and the heart; delirium and the brain; infections and the immune system. Yet, we know that advanced age, physiological stress, and infection all increase sympathetic and decrease parasympathetic nervous system function. Parasympathetic function is mediated through the vagus nerve, which connects the heart, brain, and immune system to form, what we have termed, the brain–heart-immune axis. We hypothesize that this brain–heart-immune axis plays a critical role in surgical recovery among older adults. In particular, we hypothesize that the brain–heart-immune axis plays a critical role in the most common surgical complication among older adults: postoperative delirium. Further, we present heart rate variability as a measure that may eventually become a multi-system vital sign evaluating brain–heart-immune axis function. Finally, we suggest the brain–heart-immune axis as a potential interventional target for bio-electronic neuro-immune modulation to enhance resilient surgical recovery among older adults.

## Introduction

In the next 5 years, one out of every 7 Americans age 65 and older will need major surgery, namely an invasive, non-endoscopic procedure requiring general anesthesia (Becher et al. 2023). However, major surgeries increase risk for mortality fourfold and convey a 13.4% one-year postsurgical mortality rate in frail older adults (Gill et al. 2022). Besides mortality, up to 40% of older surgical patients experience postoperative delirium (POD) (Austin et al. 2019), which increases the risk for Alzheimer's disease (AD) more than eightfold (Davis et al. 2012; Davis et al. 2017) and mortality risk up to fourfold (Hamilton et al. 2017). Yet, most surgeries are planned in advance, providing an opportunity to assess and bolster the patient’s physiological reserve and resilience. Here we propose a multi-organ system framework, centered around the vagus nerve, that underlies autonomic reserve and resilience, and we present bio-electronic medicine tools that can assess and affect multiple, interacting organ systems.

Many studies of preoperative risk factors in older adults have focused on single-organ systems, particularly the cardiovascular (CV) system and the central nervous system (CNS). However, these systems do not operate in isolation; thus, interactions among organ systems are likely a critical (and potentially modifiable) contributor to perioperative risk among older adults. Here we propose that to better understand potentially modifiable perioperative risk factors in older adults, we must understand the interactions among these organs systems, particularly the brain, the heart, and the immune system. The vagus nerve links the brain, the heart, and the immune system, thus, providing an empirical basis for a shared framework across these organ systems. Of note, diminished vagal tone—reflected by low heart rate variability (HRV)—is associated with normal aging (Chen et al. 2021), diminished cognition (Arza et al. 2015; Forte et al. 2019; Sarlija et al. 2021), poor resolution of inflammation (Aronson et al. 2001; Lampert et al. 2008), CV complications (Ernst et al. 2021), chronic illness (Christensen et al. 2021), and overall poor surgical outcomes (Ernst et al. 2017).

Given that cognitive dysfunction is the most common postoperative complication among older adults (Rudolph and Marcantonio 2011) and that the immune function required to modulate post-operative inflammation is already diminished in older age (Rea et al. 2018; Wang et al. 2020), it is possible that low vagal tone may contribute to POD pathogenesis in older adults via an unfavorably shifted BHI-A. Therefore, *we hypothesize that the heart-brain-immune axis (BHI-A) plays a crucial role in resilient surgical recovery among older adults. Further, we hypothesize that the BHI-A can be assayed *via* HRV and targeted for bio-electronic neuro-immune modulatory interventions to enhance post-operative surgical recovery and improve outcomes in older adults*.

### The Brain–Heart-Immune Axis (BHI-A)

**Fig. 1:**
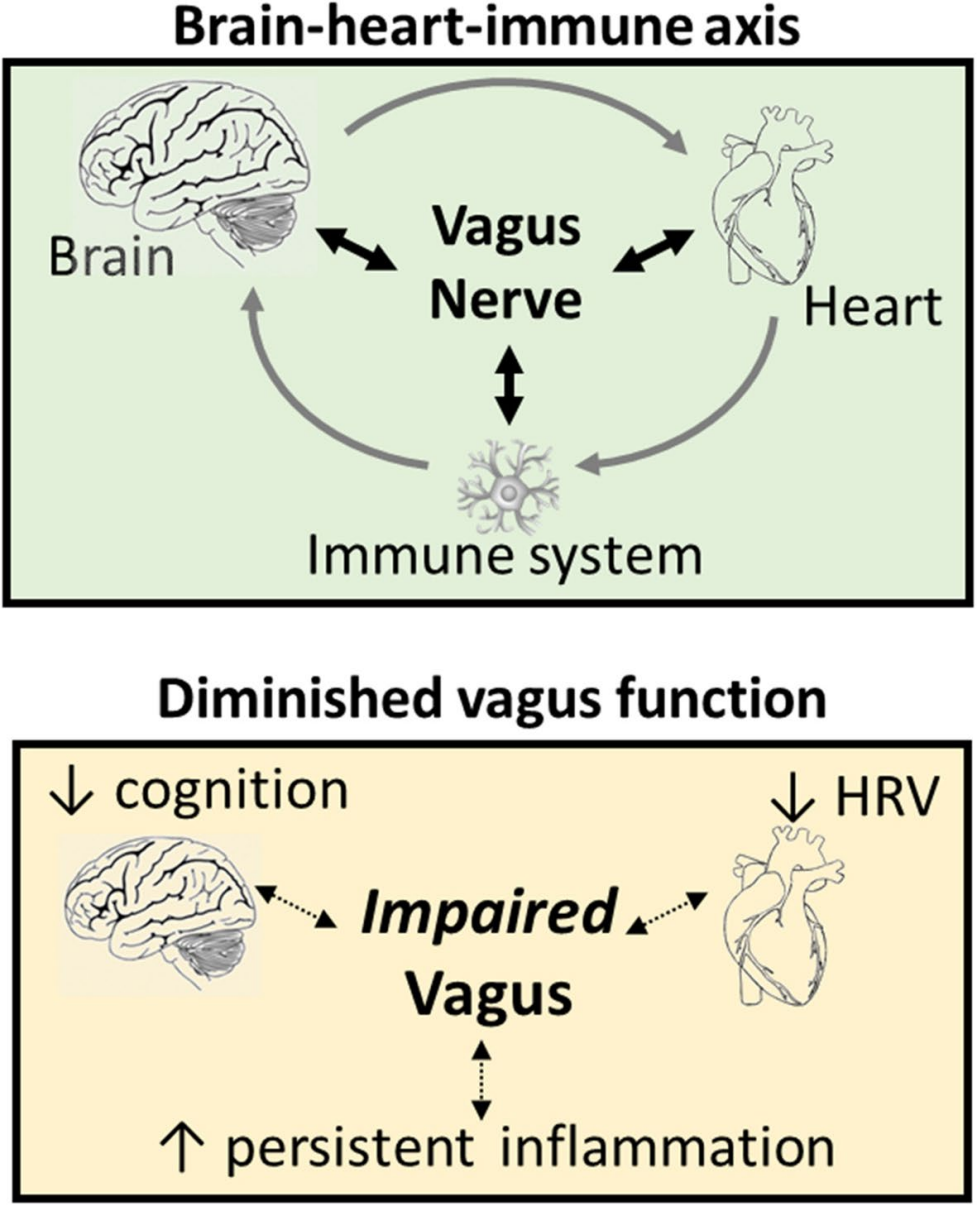
Top panel shows how the brain, heart, and immune system are connected to one another through the vagus nerve. Bottom panel shows the effects of functional vagus nerve impairment on the respective organ systems

We initially conceived the BHI-A as a multi-organ system framework to study potential mechanisms underlying POD and post-operative inflammation in older adults (Fig. 1). The BHI-A comprises three arms that link the brain, heart, and immune system to one another functionally via the autonomic nervous system (ANS) and physically via the vagus nerve and its branches. Thus, the BHI-A is a “vago-centric” model of physiological reserve and resilience.

#### The brain–heart arm

The brain–heart arm of the BHI-A was first reported in the mid-1980s by a cardiologist, Natelson, who described the role of the brain in the development of cardiac arrhythmias and sudden cardiac death (Davis and Natelson 1993; Natelson 1985), and by two experimental physiologists, Levy and Martin, who described neural control of the heart by the ANS (Levy and Martin 1984). These brain–heart hypotheses grew out of experiments showing that electrical vagus nerve stimulation (VNS) resulted in direct and reflex cardiac bradyarrhythmias (Hageman et al. 1975) and that sinus arrhythmia—a manifestation of HRV—was a reliable index of vagal cardiac outflow (Eckberg 1983). The World Stroke Organisation Brain and Heart Task Force estimates 1.5 million annual deaths result from dysfunction in the brain–heart arm of the BHI-A, largely in the setting of major adverse neurological events such as stroke, which precipitate fatal cardiac events (Sposato et al. 2020), and cardiac arrhythmias—particularly atrial fibrillation—is a leading cause of stroke (Wolf et al. 1991; Marini et al. 2005). Small, covert perioperative strokes may occur in up to 7% of non-cardiac surgery patients aged 65 and older. These small covert perioperative strokes may contribute to POD (Neuro 2019), and periventricular white matter changes—a functional magnetic imaging signature of pre-existing neurovascular disease—may predispose to cognitive dysfunction after surgery as well (Browndyke et al. 2017). The role of the ANS in these post-operative complications, however, is largely unexplored.

Even in young “healthy” populations, the brain–heart arm of the BHI-A impacts cognitive performance, particularly during emotional stress. Diminished HRV has been linked to both acute (Dimitriev et al. 2016) and chronic (Arza et al. 2015; Chalmers et al. 2014) anxiety while high HRV for age has been associated with better attentional control (Hansen et al. 2003; Luque-Casado et al. 2016; Park et al. 2012), cognitive flexibility (Alba et al. 2019), and sleep patterns (Tsai et al. 2021; Yang et al. 2011). Thus, cognitive phenotypes originating in the brain are linked to the heart through the BHI-A.

#### The brain-immune arm

**Fig. 2:**
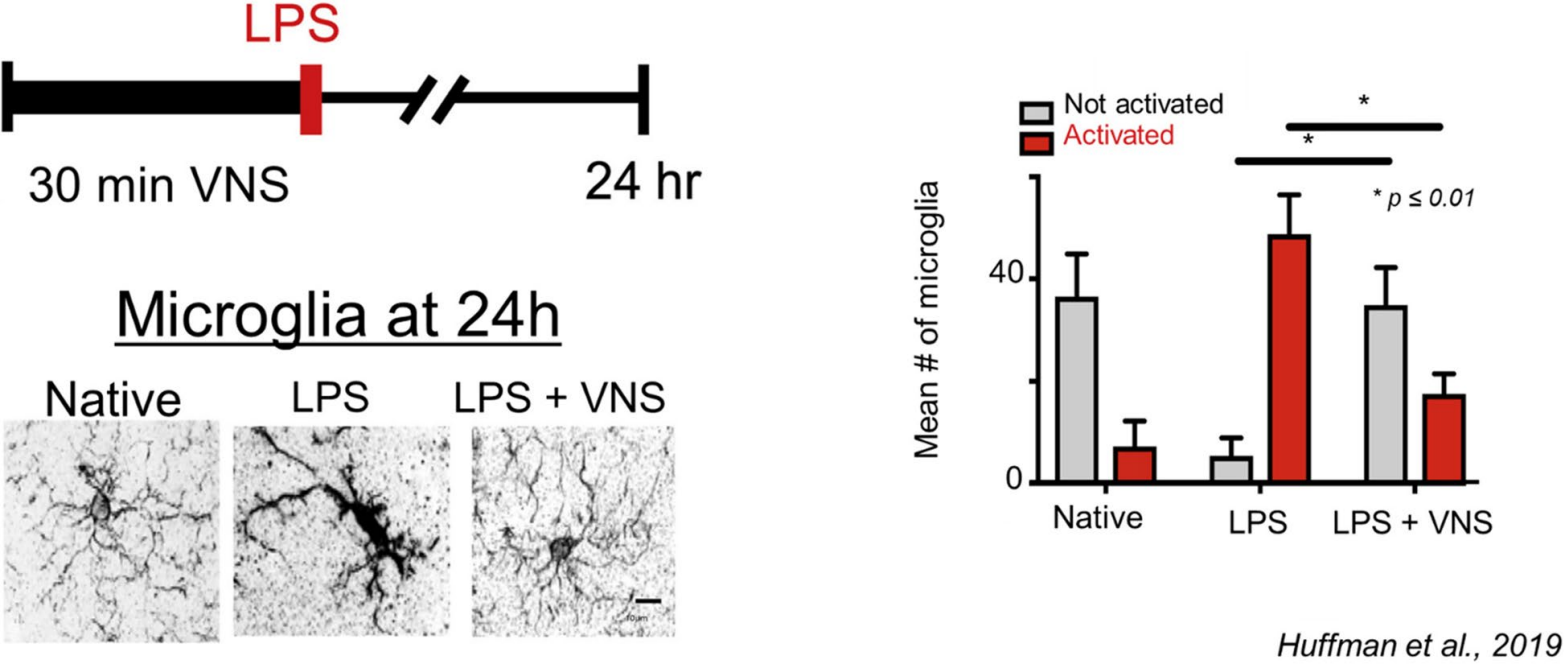
Reprinted and modified from Huffman et al., 2019. Top left panel: Lipopolysacchirde (LPS) was administered after mice received 30 min of percutaneous vagus nerve stimulation (VNS). Mice were sacrificed for histology 24 h later. Bottom left panel, while microglial were largely activated after LPS administration (middle sub-panel), when VNS preceded LPS administration microglial morphology (right sub-panel) resembled that of naïve microglia (left sub-panel). Right panel: the combination of LPS and VNS largely rescued the microglial activation resulting from LPS administration

**Fig. 3:**
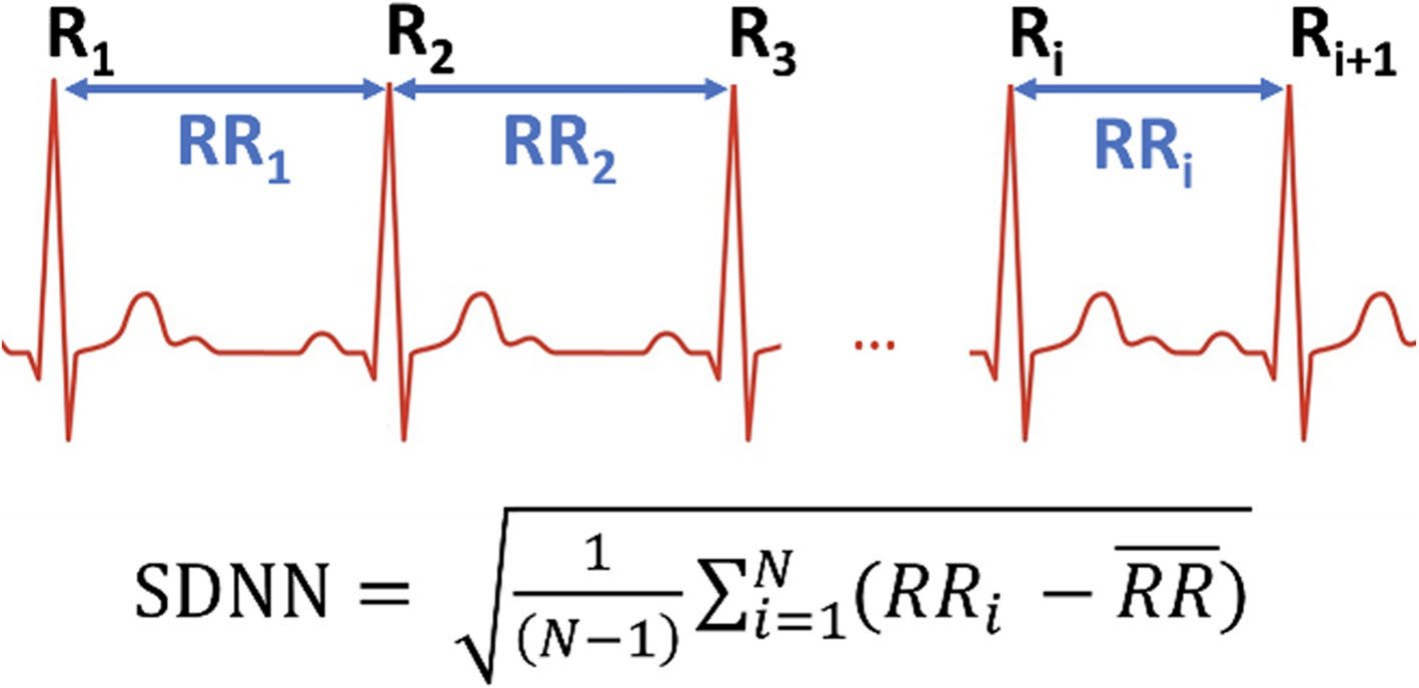
Standard deviation of normal R-R intervals (SDNN) is one of many measures of heart rate variability. SDNN, illustrated here, is sometimes used because it is easy to comprehend. HRV measures have cardiovascular physiological interpretations and are widely considered to be good indicators of vagal tone. More detail is presented in the text

The brain-immune arm of the BHIA, as first described by Tracey and colleagues (Borovikova et al. 2000; Rosas-Ballina and Tracey 2009; Tracey 2002), forms the cholinergic anti-inflammatory reflex, a parasympathetic ANS means by which the brain can reduce systemic inflammation through the vagus nerve. Pre-clinical models suggest that the cholinergic anti-inflammatory reflex also controls neuro-inflammation (Frasch et al. 2016), possibly through enhanced blood–brain-barrier (BBB) integrity (Yang et al. 2018; Lopez et al. 2012) or diminished microglial activation (Huffman et al. 2019). Beyond controlling inflammation, brain-immune arm may also limit neural injury. For example, in animal models of traumatic brain injury (TBI), which induces a “sterile immune reaction” with elevated systemic white blood cell levels, unilateral vagotomy prior to induced TBI resulted in fewer B cells and fewer CD4 + , CD25 + , and CD8 + T-cells after TBI compared with vagally intact animal models (Soares et al. 1995; Newell-Rogers, et al. 2022). Translational studies in human patients are needed to understand the potential clinical impact of these white-blood cell changes more fully. Finally, pre-clinical studies suggest that VNS may rebalance the BHI-A. Huffman et al. induced sepsis in mice via lipopolysaccharide (LPS) and then administered either VNS or sham (Huffman et al. 2019). VNS administration reversed LPS-induced microglial activation (Figs. 2 and 3), which suggests a possible role for bio-electronic medicine, particularly VNS, to modulate the brain-immune arm of the BHI-A by limiting neuro-inflammation, and possibly neural injury, which are hypothesized to underlie POD (Subramaniyan and Terrando 2019).

#### The heart-immune arm

Evidence of a vagus nerve-mediated heart-immune arm of the BHI-A has emerged from studies on cardiac healing. In particular, in pre-clinical induced acute myocardial infarction (MI) models, treatment with pyridostigmine, a cholinesterase inhibitor, has been shown to (1) increase parasympathetic tone, as measured by HRV and baroreceptor sensitivity (Bandoni et al. 2021; Barboza et al. 2019); (2) to induce a more favorable, anti-inflammatory M1/M2 macrophage profile (Bandoni et al. 2021; Rocha et al. 2016); (3) to diminish levels of inflammatory cytokines in the heart’s ventricles (Barboza et al. 2019) and (4) to limit pathologic post-MI ventricular remodeling, which better preserves post-MI left ventricular ejection fraction (Bandoni et al. 2021). Thus, while heart-immune interactions remain an active research area with more investigation in human patients needed, current evidence suggests that the heart and immune system interact to promote overall homeostasis and healing (Thayer and Fischer 2009; Williams et al. 2019; Weber et al. 2010; Cooper et al. 2015).

In sum, the ANS functionally links the CNS, the CV and immune systems to form the vagus nerve-mediated BHI-A. The BHI-A allows an integrated, holistic framework to study the effects of physiological derangements such as illness, injury and surgery across multiple organ systems and is likely particularly valuable for studying reserve and resilience in older surgery patients.

##### Heart rate variability (HRV) as a surrogate measure of vagal tone and resilience

HRV has become the standard surrogate measure for vagal tone (Shaffer and Ginsberg 2017; Adamson et al. 2004; Anderson 2017; Grote, et al. 2019) in part because it is non-invasive and simple to collect compared to alternative methods (Laborde et al. 2017). HRV measurement is a sensitive indicator of parasympathetic health, and allows for nuanced evaluation of autonomic nervous system (ANS) flexibility. HRV measurement uses a combination of time and frequency domain variables, as reviewed by Shaffer and Ginsberg (Shaffer and Ginsberg 2017). Time domain measures inform the amount of variability of the inter-beat-interval (IBI) while frequency domain measurements describe power distribution across ultra-low- (ULF, ≤ 0.003 Hz), very-low- (VLF, 0.003–0.04 Hz), low frequency (LF, 0.04–0.15 Hz) and high frequency (HF, 0.15–0.4 Hz) bands (Shaffer and Ginsberg 2017). LF and HF band and peak power values have been widely studied and were initially assumed to reflect sympathetic and parasympathetic ANS activity respectively; however, it is now known that LF power is an admixture of many different ANS factors (Billman 2013). A spectral power peak at 0.1 Hz during slow breathing at rest is understood to closely reflect indicate baroreceptor activity (McCraty and Shaffer 2015). The HF band, which corresponds to frequencies of respiration and so is sometimes called the ‘respiratory band,’ has been correlated with cardiac parasympathetic activity with a strong direct correlation between HF power and direct recording of cardiac parasympathetic fibers in animal models (Piccirillo et al. 2009). Even simple time domain HRV measures vary with age (Umetani et al. 1998), sex (Christensen et al. 2021; Shaffer and Ginsberg 2017; Umetani et al. 1998), and race (Lampert et al. 2005; Wang et al. 2005) underscoring the need for more robust normative HRV data from diverse, well-defined, and appropriately sampled populations.

Across different occupational and demographic groups, high vagal tone is important for adapting to acutely stressful situations. For example, those with stressful occupations—such as firefighters, air traffic controllers (Sarlija et al. 2021) and police officers (Weltman et al. 2014; Whitson et al. 2021), tend to have higher resting HRV reflecting a more robust vagal system. Just as maintaining cardiovascular homeostasis in situations of physical and psychological environmental stress is challenging for those with stressful occupations, maintaining homeostasis is critical in the face of surgical trauma, particularly among older adults undergoing surgery who may have lower physiological resilience. Environmental stress and surgery both require similar adaptive cardiovascular, cognitive, and inflammatory responses (Whitson et al. 2021). Recovery from surgical trauma and favorable surgical outcomes in younger (Caton et al. 2021) and older patients (Ernst et al. 2021; Ernst et al. 2017; Echizen et al. 2021) are associated with higher HRV (Ernst et al. 2021; Ernst et al. 2017; Caton et al. 2021; Echizen et al. 2021). Therefore, we hypothesize that those with higher resting HRV pre-operatively will be more resilient to surgical trauma and recover faster. Further, pre-stress vagal conditioning, through active interventions such as training or simply through healthy lifestyle choices, can attenuate psychological and physiological stress reactions and, thus, promote resilient recovery through parasympathetic health (Gharbo 2020).

Given that HRV is an accessible, validated indicator of vagal tone and parasympathetic health, we hypothesize that HRV will continue to be a useful, physiologically relevant measure of BHI-A function in coming years for several reasons. First, technological advances will allow for normative data collection and advanced HRV measures with even closer links to ANS function, such as the ubiquity of wearable devices, increased computing power, new signal processing techniques, and rapid advances in artificial intelligence. Second, these powerful new tools allow continuous HRV data collection over days, weeks or longer. Finally, multi-organ system studies—such as our “HRV in POD and post-operative inflammatory endpoints” (HiPPIE) study briefly described below—provide data from multiple organ systems to relate back to HRV data. Long duration, multi-organ system measurements encompassing diurnal variations and sleep cycles provide powerful, ecologically valid conditional manipulations. These natural manipulations will provide greater insight into ANS function throughout the normal course of life and recovery from illness and injury. In turn, these measurements and techniques will allow us to assay BHI-A function longitudinally among patients who recover resiliently and those who do not.

##### The BHI-A in older surgical patients

Older adults, particularly those with dementia (Toledo and Junqueira 2008; Nicolini et al. 2020), manifest lower HRV than younger adults (Chen et al. 2021; Umetani et al. 1998; Dalise, et al. 2020), which reflects the relative increase in sympathetic versus parasympathetic ANS activity with aging. For older surgery patients, advanced age, the condition requiring surgery, and preoperative stress, are all associated with lower HRV measures (Umetani et al. 1998; Dalise, et al. 2020) that reflect a shift in the balance between sympathetic and parasympathetic ANS activity even further toward sympathetic activation. Consistent with non-surgical populations in which low HRV is associated with poor cognitive performance (Forte et al. 2019; Luque-Casado et al. 2016; Blons et al. 2019), inflammation (Lampert et al. 2008; Borovikova et al. 2000; Tracey 2002) and diminished immune function (Borovikova et al. 2000; Koopman et al. 2016; Saeed et al. 2005). A study of older hip fracture patients suggested those who developed post-operative delirium had higher low frequency (LF) and higher high frequency (HF) HRV values prior to surgery (Ernst et al. 2020); however, these data were difficult to interpret because nearly half of hip fracture patients presented with delirium prior to surgical repair and because all had sustained a major injury prior to HRV evaluation. Therefore, additional studies of pre-operative HRV in older adults before major elective surgery are needed.

### Vagus nerve stimulation as supporting evidence for our hypothesis

As supporting evidence for our hypothesis, we note that VNS may have potential to modulate neuro-inflammation and cognition via the BHI-A.

Pre-clinical studies suggest that VNS may rebalance the BHI-A, as illustrated in the aforementioned Huffman et al. (Huffman et al. 2019) study. Here, VNS administration reversed lipopolysaccharide (LPS)-induced microglial activation in mice (Fig. 2), suggesting a possible role for bio-electronic medicine, particularly VNS, to modulate the brain-immune arm of the BHI-A by limiting neuro-inflammation and neural injury, which are hypothesized to underlie POD (Yang et al. 2020). Mice treated with VNS not only had a more favorable microglial profile, but they also achieved a 10% reduction in heart rate and a factor recovery of cognitive performance post-surgery (Huffman et al. 2019).

Further, human studies show promise for VNS-induced immune normalization. Nine patients with moderate Crohn’s disease underwent continuous left VNS (Sinniger et al. 2020) via implanted circumferential electrode for one year. At the end of the year, five patients were in clinical remission and six were in endoscopic remission. Seven patients restored their vagal tone to near homeostatic levels, and, in general, the patients’ cytokine profiles showed more normal character. In another study, 20 patients with rheumatoid arthritis (RA) underwent transcutaneous VNS with resulting in reductions in RA-associated biomarker levels (Drewes et al. 2021) consistent with anti-inflammatory effects of VNS. Overall, VNS shows promise for modulating the BHI-A such that VNS may eventually be used to enhance physiological resilience in the face of acute stressors, such as surgery.

In addition to electrical VNS, we note that non-pharmacological therapies that increase vagus tone—such as slow deep breathing and mindfulness meditation—have been employed for thousands of years. Indeed, the BHI-A is a concept that helps to explain the modern benefits of these ancient practices. Further, the advent of wearable physiological monitors also advances non-drug, non-electrical techniques to bolster BHI-A function. For example, cardiac coherence uses biofeedback from wearable monitors to coordinate breathing with the heartbeat and, thereby, increase vagal tone.

### HRV in POD and Postoperative Inflammatory Endpoints (HiPPIE)

As a first step toward understanding the role of the BHI-A in surgical recovery and its potential as an interventional target, the HiPPIE (HRV in POD and Post-operative Inflammatory Endpoints) study will enroll up to 150 Duke patients age 65 and older undergoing scheduled, non-cardiac, non-intracranial surgeries. HiPPIE, a Duke Health Institutional Review Board approved study provides a unique opportunity to evaluate the role of vagal tone as an indicator of POD risk before and immediately after surgery. The HiPPIE study evaluates interactions among the organ systems of the BHI-A using (a) wearable devices, (b) pre-operative and twice daily post-operative delirium assessments with the 3-min confusion assessment method (3D-CAM: Derivation and Validation of a 3-Minute Diagnostic Interview for CAM-Defined Delirium. 2014), and (c) serial plasma biomarker samples to measure inflammation before surgery and 24-h and 48-h after surgery.

HiPPIE measures continuous HRV via age-friendly, convenient wristbands (Corsano B.V) which have no watch face and battery life up to 7 days. Patients are instructed simply to charge the wristband while swimming, bathing, or showering and to wear the wristband at all other times. By capturing HRV from the time of the pre-operative evaluation (typically 5–15 days before surgery) through the second day after surgery, when POD incidence peaks (Robinson et al. 2009), we expect HiPPIE to capture a unique dataset encompassing the perioperative function across multiple, interacting BHI-A organ systems. In addition, HiPPIE has a sister pilot feasibility study of perioperative transcutaneous VNS in a similar population, which we call POTENT (Pre-Operative Transcutaneous auricular vagus nerve stimulation Effects on Neuro-inflammatory Trends). POTENT is also a Duke Health Institutional Review Board Approved study.

## Conclusion

We present a novel multi-organ system framework, the BHI-A, as a tool for understanding perioperative resilience to post-operative delirium, and we introduce HRV and vagal nerve stimulation as means to measure and modulate the BHI-A, respectively. We fully expect that the BHI-A framework could be applied to many other disorders and that HRV and VNS are new bio-electronic means of exploring and enhancing resilience. Prior studies in humans and animals offer compelling evidence that interventions targeting vagal tone modulation may offer substantial benefits for controlling systemic inflammation and neuro-inflammation, which are believed to contribute to POD as well as many other neurocognitive disorders, such as Alzheimer’s Disease. Further, our studies advance the use of the BHI-A framework in understanding and modulating perioperative reserve and resilience, and we would advocate that viewing common disorders through the BHI-A framework would afford more opportunities for bio-electronic monitoring and bio-electronic interventions to advance human health broadly.

## Data Availability

No new data or materials are presented in this manuscript.

## Abbreviations

AD: Alzheimer’s Disease
ANS: Autonomic Nervous System
BHI-A: Brain–Heart-Immune Axis
CNS: Central Nervous System
CV: Cardiovascular
HiPPIE: HRV in POD and Post-operative Inflammatory Endpoints
HRV: Heart rate variability
LPS: Lipopolysaccharide
POD: Post-operative delirium
POTENT: Pre-Operative Transcutaneous auricular vagus nerve stimulation Effects on Neuro-inflammatory Trends
SDNN: Standard deviation between normal-normal beats
VNS: Vagus Nerve Stimulation
